# Fundus Albipunctatus Associated with Biallelic LRAT Gene Mutation: A Case Report with Long-Term Follow-Up

**DOI:** 10.3390/jcm12226960

**Published:** 2023-11-07

**Authors:** Wendy D. Tan, J. Vernon Odom, Monique Leys

**Affiliations:** Department of Ophthalmology and Visual Sciences, West Virginia University School of Medicine, Morgantown, WV 26506, USA; jodom@hsc.wvu.edu (J.V.O.); leysm@wvumedicine.org (M.L.)

**Keywords:** fundus albipunctatus, congenital stationary night blindness, LRAT gene, inherited retinal disorder

## Abstract

This case report presents a 26-year-old female patient diagnosed with fundus albipunctatus (FAP), a rare form of congenital stationary night blindness. The patient’s clinical history and retinal findings spanning 23 years are consistent with FAP. The patient has profound night blindness, photophobia, and mild color vision changes with preserved best-corrected visual acuity (BCVA). Small white dots are present throughout the fundus, sparing the central macula. Electroretinograms (ERG) are consistent with congenital stationary night blindness (CSNB) and suggest a lack of rod response. Ophthalmic imaging has remained stable over time. Genetic testing revealed two biallelic missense mutations in the LRAT gene, c.197G>A (p.Gly66Glu) and c.557A>C (p.Lys186Thr). LRAT mutations are known to contribute to other retinal conditions but have not been previously associated with FAP. While there are currently no available treatments for FAP, this report expands our understanding of the genetic landscape of FAP to include LRAT and provides clinical data to support this finding.

## 1. Introduction

Fundus albipunctatus (FAP, OMIM #136880) is a rare inherited retinal disease that causes a form of congenital stationary night blindness. FAP is characterized by the presence of small, white or yellow-white, punctate lesions throughout the periphery at the level of the retinal pigment epithelium (RPE). Night blindness and functional visual deficits in FAP are not progressive, rather they remain relatively stable throughout the affected person’s life. The disease presents in early childhood and predominantly affects rod photoreceptors.

FAP is primarily associated with gene mutations in retinol dehydrogenase 5 (RDH5) and retinaldehyde binding protein 1 (RLBP1) in an autosomal recessive inheritance pattern [1,2,3]; however, there have been reports of FAP associated with heterozygous RPE65 single and compound mutations [4,5]. RDH5, RPE65, and RLBP1 encode proteins expressed in the RPE involved in the visual (retinoid) cycle. Lethicin retinol acyltransferase (LRAT) is another gene involved with the visual cycle that encodes an enzyme expressed in the RPE that we propose could also be linked to FAP.

In the RPE, a series of enzymatic reactions regenerate rhodopsin in the visual cycle, where the proteins encoded by RDH5, RPE65, and LRAT each mediate a step in the conversion of all-trans-retinol to 11-cis-retinal. RLBP1 encodes cellular retinaldehyde-binding protein that binds, transports, and stabilizes retinoids in the RPE as they progress through this pathway. Defects in these visual cycle enzymes lead to impaired rhodopsin regeneration and prolonged photoreceptor desensitization to light that result in the night blindness symptoms of FAP [6]. Furthermore, it is hypothesized that the hyperautofluorescent white dots visualized in FAP are toxic retinoid accumulations in the RPE, which has been shown in RDH5 knockout mouse models [7,8].

LRAT mutations are implicated in multiple phenotypic presentations, such as Leber congenital amaurosis, retinitis punctata albescens, and retinitis pigmentosa [9,10,11]. To our knowledge, LRAT has not previously been associated with FAP in the medical literature; however, since LRAT operates in conjunction with established genes that cause FAP, we report a case that proposes mutations in LRAT can be linked to FAP. In this case report, we characterize a patient diagnosed with FAP, whose genetic testing implicates two LRAT variants of unknown significance. This patient has 23 years of follow-up ophthalmic assessment and imaging to support this diagnosis.

## 2. Clinical Presentation

A 26-year-old female patient with congenital night blindness has been followed since age 3 into adulthood. She experiences night blindness, delayed dark adaptation, and severe photophobia.

At age 3, the patient presented with visual defects and night blindness. The patient’s parents had concerns about ocular misalignment and inability to see in the dark. She had met typical developmental milestones for her age. Upon the patient’s initial assessment, her fixation was central–steady–maintained and did not have any ocular alignment defects in any gaze position with full ocular movement. The patient’s best-corrected visual acuity (BCVA) was 20/40 in each eye. Cone-adaptation testing (Good-Lite) showed an inability to dark adapt [12]. Electroretinogram (ERG) testing under anesthesia was completed with the standard operating room protocol using the Retinographics BPM-100 system and Jet Contact lens electrodes. Flash ERGs were recorded after light adaptation from the left eye and after a 20 min dark adaptation from the right eye. Light-adapted results were roughly within normal limits for amplitudes but appeared delayed and dark-adapted results were reduced in amplitude but within normal latency. The dark-adapted response was similar to the light-adapted response, suggesting the possibility of a lack of rod response and only a cone response. This ERG at age 3 was interpreted as consistent with congenital stationary night blindness. Lea symbol testing noted normal contrast sensitivity. The patient’s color vision assessment using Hardy–Rand–Rittler plates was normal at age 5.

**Figure 1:**
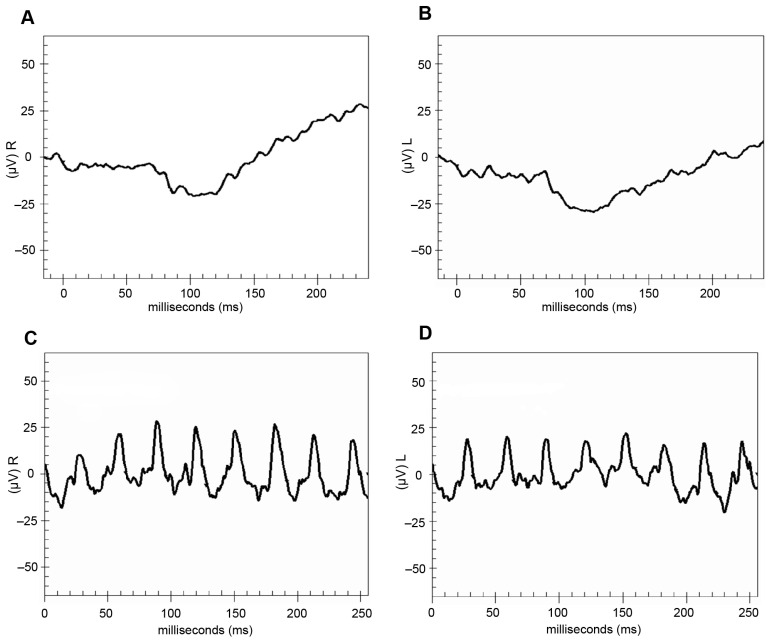
Electroretinography (ERG) and adaptometry testing. (**A**–**D**) ERG performed at age 7 using LKC Utas user-defined protocol based on the 2008 ISCEV standards and skin–electrodes [13]. The flash intensity for the light-adapted and dark-adapted conditions presented was 2.5 cd/s/m^2^. Background for the light-adapted condition was 30 cd/m^2^. (**A**) Right eye, dark-adapted flash ERG. (**B**) Left eye, dark-adapted flash ERG. (**C**) Right eye, 30 Hz flicker ERG. (**D**) Left eye, 30 Hz flicker ERG.

ERGs were repeated at age 7 binocularly using the LKC Utas user-defined protocol based on the 2008 ISCEV standard with skin–electrodes [13]. Flash ERGs were recorded in the following manner: after pupillary dilation and 20 min of dark adaptation, a skin–electrode was placed underneath the patient’s eye to record the retina’s response to a flashing light. Subsequently, the patient was light-adapted for 10 min and the retina’s response to flashing lights was recorded. The use of the skin–electrode tends to attenuate the response by a factor of 5–10 but does not affect response timing. The standard flash intensity was 2.5 cd/s/m^2^ with a background light of 30 cd/m^2^. Results were consistent with nyctalopia. The cone pathways appeared relatively well preserved as the 30 Hz response appears to reflect primarily cone activity based on the similarity in amplitude and timing of the cone and mixed responses (Figure 1).

At age 9, the patient’s BCVA at distance was stable at 20/25 in each eye, but was reduced to 20/70 in each eye under conditions of reduced illumination. Her cycloplegic refraction was +2.50 + 2.00 × 90 OD, +2.00 + 0.75 × 90 OS.

Genetic testing was conducted through the Prevention Genetics Inherited Retinal Disorders Panel (31 genes) when the patient was 20 years old and repeated by the Invitae Inherited Retinal Disorders Panel (330 genes) when the patient was 25. The results of both were consistent and positive for two heterozygous missense LRAT mutations of uncertain significance, c.197G>A (p.Gly66Glu) and c.557A>C (p.Lys186Thr). A heterozygous missense mutation CEP290 c.7430C>G (p.Pro2477Arg) of uncertain significance was also found. Additionally, a heterozygous intronic RLBP1 mutation (c.684 + 20C > T) was identified; however, it is classified as benign and likely does not contribute to her retinopathy. Her genetic testing was negative for RDH5 and RPE65 mutations. Genetic testing of the patient’s biological mother and father determined the contribution of the c.557A>C mutation from the father and c.197G>A mutation from the mother, thus confirming the LRAT mutations are biallelic. Provided that there were no pathogenic variants detected and no other plausible genetic causes to her phenotype, it was deduced that the biallelic LRAT mutations were the cause of FAP in the patient.

**Figure 2:**
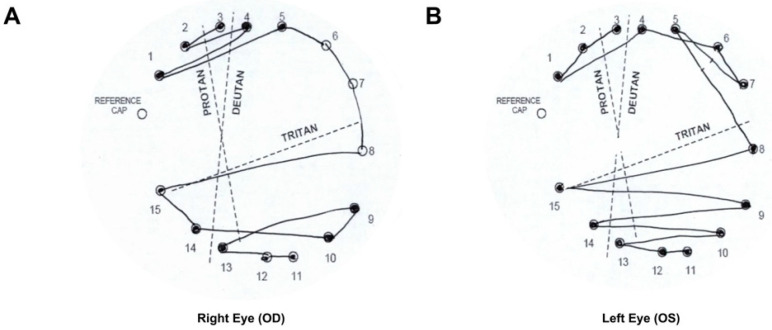
D-15 color vision assessment at age 21 shows color vision defects in both eyes. (**A**) Right eye. Tritan errors, indicating predominantly blue-yellow color vision changes. (**B**) Left eye. Tritan and protan errors, indicating mild blue-yellow and mild red-green color vision changes, respectively.

At age 21, ERG with RETeval (LKC Technologies, Gaithersburg, MD, USA) showed a poor scotopic response, present photopic response, and delayed decreased flicker response. However, the patient’s severe photophobia and difficulty tolerating prolonged dark adaptation limited the quality of the adaptometry and ERG testing data. A mild tritan color vision deficit in both eyes was noted with a D-15 color vision assessment with some additional protan errors in the left eye (Figure 2). Of note is that the patient’s serum vitamin A level was 55.8 mcg/dL, within the normal range of 32.5–78 mcg/dL.

**Figure 3:**
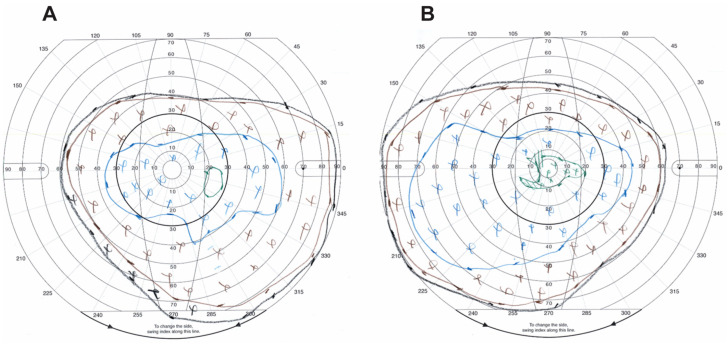
Goldmann visual field at age 26. Black (V4e), brown (III4e), blue (I4e), and green (I3e) isopters shown. “X” denotes individual points seen by the patient at each stimulus level of corresponding color. (**A**) Right eye. (**B**) Left eye.

At age 26, the patient’s best-corrected visual acuity (BCVA) remains stable at 20/20 in each eye (−1.50 + 3.25 × 90 OD, −2.25 + 3.50 × 95 OS). She has reported no subjective changes to her vision. Goldmann visual field testing shows no peripheral visual constriction with I4e and a peripheral field that has remained relatively stable over time (Figure 3). She has obtained a driver’s license with her visual field and acuity, although she is unable to drive at night.

**Figure 4:**
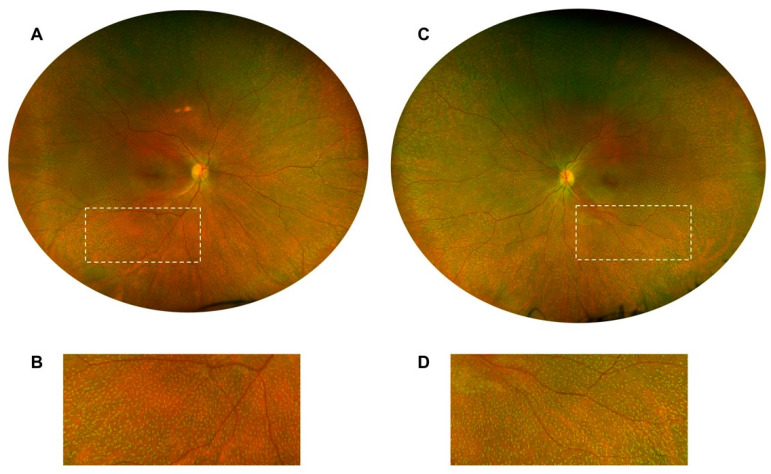
Widefield color fundus photography images, showing 360 degrees of fine white dots throughout the fundus in both eyes, fovea sparing. Images taken at age 26 on the Optos California retinal imaging device (Optos, Dunfermline, Scotland, UK). (**A**) Right eye, full fundus image. (**B**) Magnification of right eye peripheral area, corresponding to white dashed box, shows numerous white dots of fundus albipunctatus (FAP). (**C**) Left eye, full fundus image. (**D**) Magnification of left eye peripheral area, corresponding to white dashed box, shows numerous white dots.

**Figure 5:**
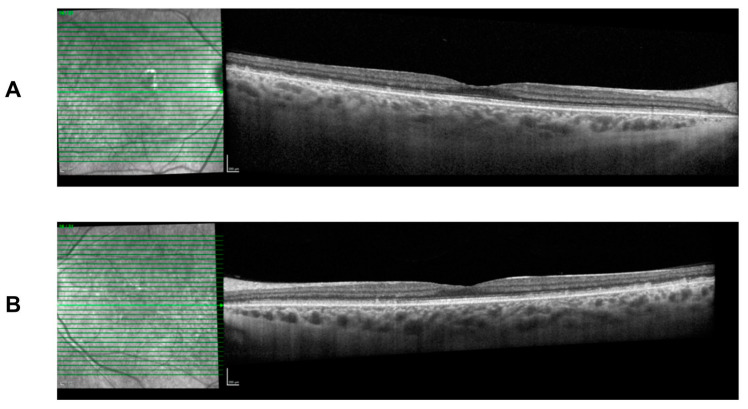
Spectral-domain optical coherence tomography (SD-OCT) at age 26 with deep retinal deposits that are fovea sparing. There is mild attenuation of the ellipsoid zone and a thick choroid. Findings are symmetrical in both eyes. (**A**) Right eye. (**B**) Left eye.

She continues to present with the typical FAP phenotype of small subretinal white dots 360 degrees throughout the fundus, particularly in the mid-periphery with fundoscopy and retinal imaging (Figure 4). The white dots spare the fovea region and preserve her visual acuity. Spectral-domain optical coherence tomography (SD-OCT) imaging is stable over time and shows the presence of subretinal deposits, attenuated ellipsoid zones, and a thick choroid in both eyes (Figure 5).

## 3. Discussion

In this case report, we present a patient with FAP, supported by 23 years of follow-up ophthalmic assessments from ages 3 to 26, that implicates two biallelic mutations in the LRAT gene in her disease. This is likely one of the first reported associations between FAP and the LRAT gene, adding to the list of reported genetic mutations implicated in FAP, which have predominantly been within the RDH5 and RPE65 genes in the current literature.

Throughout follow-up assessments and imaging, the natural history and progression of the patient’s FAP has shown a stable clinical phenotype. Nyctalopia and light vision deficits have subjectively remained stable with delayed dark adaptation. The patient’s best-corrected visual acuity continues to remain stable over 7 years; however, the patient’s color vision assessments indicate there may have been a progression of color vision deficits between the ages of 5 and 21. OCT imaging has remained stable over time, showing a lack of fovea involvement and little impact on central vision.

The identification of these novel LRAT gene mutations in our patient expands our understanding of the genetic basis of FAP. Currently, there is limited knowledge on the two heterozygous missense LRAT mutations with which our patient presents, c.197G>A (p.Gly66Glu) and c.557A>C (p.Lys186Thr). Both mutations are classified as variants of uncertain significance due to a lack of mutation-disease associations in the literature and inconclusive predictive algorithms on the protein structural/functional consequences of these mutations [14]. Predictive algorithms suggest that the c.557A>C mutation may disrupt a consensus splice site [14]. In future studies, characterizing these specific mutations and studying the functional consequences will allow for insight into the underlying molecular mechanisms of the disease that implicate visual cycle genes expressed in the RPE involved in converting all-trans-retinal back to 11-cis-retinal. We predict the LRAT gene mutations identified in our patient likely disrupt the normal enzymatic activity of LRAT, impairing the regeneration of 11-cis-retinal and causing the clinical manifestations of FAP.

The patient received orientation and mobility services for night blindness during her childhood and visual accommodations at her employment. She received counseling on lifestyle recommendations including a diet rich in antioxidants, limited UV exposure, and smoking risks. The patient’s vision will continue to be monitored to map any changes to her central or peripheral vision over time. Full-field stimulus testing (FST) may be considered in the future in addition to high-resolution imaging. Additionally, fluorescein angiography may be considered for future testing in order to visualize the punctate dots; however, the patient’s severe photophobia is a limiting factor. While the patient’s imaging and visual acuity have remained stable at her current age, we find it difficult to predict whether the visual cycle defects underlying FAP could eventually lead to the retinal and macular degeneration of photoreceptors. There is evidence that FAP can present with or without cone dysfunction and that cone dysfunction is more frequently observed in older FAP patients with RHD5 mutations [15], but long-term studies suggest clinical stability and do not suggest progressive cone or rod dysfunction [16].

Currently, there are no treatments for FAP available. A Phase 1 safety and proof-of-concept clinical trial was conducted on patients with Leber congenital amaurosis or retinitis pigmentosa due to LRAT or RPE65 mutations to determine if oral 9-cis-retinyl acetate, a 9-cis-retinal precursor, was able to improve functional visual outcomes by bypassing the toxic retinoid accumulations in the visual cycle from defective proteins encoded by either mutated LRAT or RPE65 [17,18]. The clinical trial did not progress past Phase 1; however, more research into retinoid alternatives could yield promising data in the future.

## 4. Conclusions

This case report implicates two novel biallelic LRAT gene mutations in a patient with fundus albipunctatus, a previously unreported association. These findings contribute to our understanding of the genetic basis of FAP and highlight the importance of genetic testing in the diagnosis and management of this condition. Further research is warranted to fully characterize the impact of LRAT gene mutations on visual function and explore potential therapeutic avenues for affected individuals.

## Data Availability

The data presented in this case report are available upon request from the corresponding author.
